# Empowering Communities: A Review of Community-Based Outreach Programs in Controlling Hypertension in India

**DOI:** 10.7759/cureus.50722

**Published:** 2023-12-18

**Authors:** Om Prakash Bera, Himel Mondal, Sudip Bhattacharya

**Affiliations:** 1 Health Systems Strengthening Unit, Global Health Advocacy Incubator, Washington, DC, USA; 2 Physiology, All India Institute of Medical Sciences, Deoghar, Deoghar, IND; 3 Community and Family Medicine, All India Institute of Medical Sciences, Deoghar, Deoghar, IND

**Keywords:** developing countries, india, cardiovascular diseases, primary healthcare, public health, blood pressure, non-communicable disease, private sector, community outreach programme, hypertension

## Abstract

India's epidemiological shift from communicable to non-communicable diseases (NCDs) signifies the impact of healthcare advancements and changing lifestyles. Despite declines in infectious diseases, challenges related to chronic conditions such as cardiovascular diseases and diabetes have risen. Approximately one in four Indian adults has hypertension, with only 12% maintaining controlled blood pressure. To meet the 25% relative reduction target in hypertension prevalence by 2025, India must enhance treatment access and public health initiatives. A global report underscores the urgency of preventing, detecting, and managing hypertension, especially in low- and middle-income countries like India, where 188.3 million adults are estimated to have hypertension. Loss to follow-up persists in both communicable and non-communicable diseases, driven by factors such as stigma and socioeconomic barriers. Community outreach programs have proven effective, incorporating mobile health interventions, community health worker engagement, and door-to-door screenings. Hypertension management faces similar challenges, with community outreach tailored to lifestyle factors and cultural beliefs showing promise. The comprehensive strategy to control hypertension involves strengthening primary healthcare centers, promoting wellness centers, and capacitating Community Health Officers. While community-led, tech-enabled private sector interventions can screen and manage NCDs, integration with the public health system is crucial for widespread adoption and cost-effectiveness. In conclusion, tailored strategies, such as community outreach integrated into healthcare systems, are essential to address loss to follow-up and enhance health management success in both communicable and non-communicable diseases.

## Introduction and background

In India, the epidemiological landscape has undergone a profound transition from a predominance of communicable diseases to an increasing burden of non-communicable diseases (NCDs). Historically, infectious diseases such as malaria, tuberculosis, and diarrheal illnesses posed significant public health challenges. However, with advancements in healthcare, sanitation, and vaccination programs, there has been a gradual decline in the prevalence of communicable diseases. Simultaneously, lifestyle changes, urbanization, and shifts in dietary habits have contributed to a rise in non-communicable diseases, including cardiovascular diseases, diabetes, and certain cancers. This epidemiological transition reflects improvements in public health interventions and healthcare infrastructure, but it also underscores the emergence of new challenges posed by chronic, lifestyle-related conditions. For cardiovascular disease, hypertension is one of the important risk factors for premature mortality [1].

Approximately one in four adults in India is estimated to have hypertension, yet only around 12% of them manage to maintain controlled blood pressure. In an effort to address this health challenge, India has established a target aiming for a 25% relative reduction in the prevalence of hypertension by 2025 [2]. Achieving this goal necessitates the expedited enhancement of access to treatment services, with a particular focus on strengthening public health initiatives. Given that uncontrolled blood pressure stands as a major risk factor for cardiovascular diseases (CVDs) such as heart attacks and strokes, which are globally recognized as the leading cause of death and disease, the imperative for effective hypertension control strategies is underscored. CVDs account for a significant portion of total deaths in India, amounting to approximately one-third of the overall mortality rate. Despite an estimated 220 million individuals in India living with hypertension, merely 12% have successfully maintained controlled blood pressure [3].

During the 78th United Nations General Assembly (UNGA), the World Health Organization (WHO) presented a pivotal report titled "Global Report on Hypertension: The Race Against a Silent Killer." This marked its inaugural assessment of the worldwide impact of hypertension, or high blood pressure. The report unveiled staggering figures, revealing that one in three adults globally, totaling 1.3 billion individuals between 1990 and 2019, is affected by hypertension. Despite its widespread prevalence, about four out of every five individuals with hypertension lack adequate treatment. In India, an estimated 188.3 million adults aged 30-79 are burdened with hypertension, emphasizing a significant national challenge. Achieving a 50% control rate in India necessitates effective treatment for an additional 67 million people, potentially averting 4.6 million deaths by 2040. The report emphasized global disparities in treatment coverage, with high-income nations exhibiting more favorable rates, such as the WHO region of the US leading with a 60% coverage rate, while the African region lagged at 27%. The urgency for timely intervention was highlighted, especially for the nearly 30% of individuals with uncontrolled hypertension requiring urgent measures. The report called for a comprehensive response, urging the prioritization of prevention, early detection, and effective hypertension management as integral components of national health benefit packages. It underscored the imperative to strengthen globally under-prioritized and underfunded hypertension control programs, advocating for their integration into every country's journey toward universal health coverage [4].

With this background, this study aimed to review the current problems faced in following up with patients having hypertension and how to mitigate those to strategize a roadmap for improving the control rate of hypertension in India.

## Review

Source of literature

**Figure 1:**
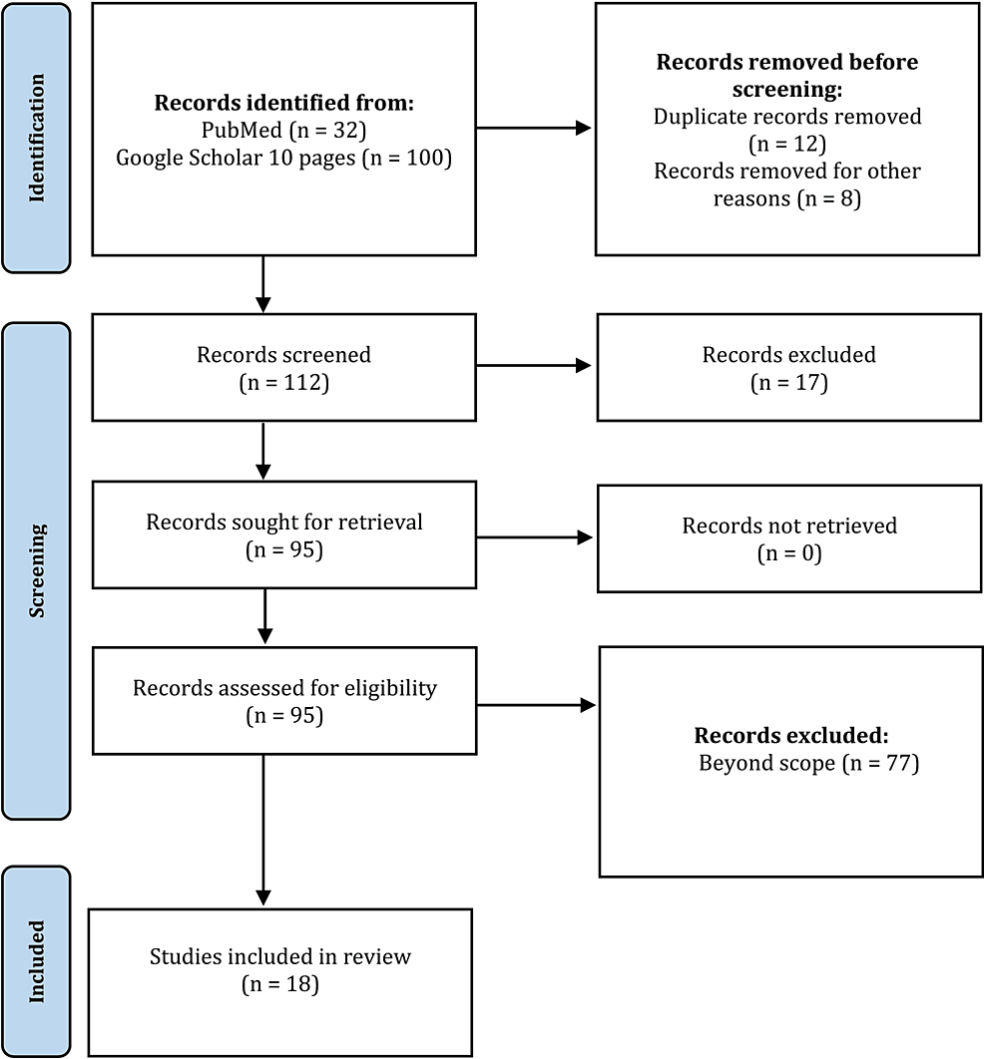
Preferred Reporting Items for Systematic Reviews and Meta-Analyses flow chart

We searched the literature in PubMed and Google Scholar with the keywords "hypertension" and "Community-Based Outreach Program" and qualitatively analyzed those for this review. The present systematic review adheres to the Preferred Reporting Items for Systematic Reviews and Meta-Analyses (PRISMA) guidelines. The PRISMA flow chart is shown in Figure 1.

The systematic review included peer-reviewed articles focusing on community-based interventions for hypertension control. Accepted study designs encompass randomized controlled trials, cohort studies, case-control studies, observational studies, and reviews. To ensure a comprehensive review, there were no restrictions on the publication date or geographic limitations.

Exclusion criteria involved non-peer-reviewed articles, interventions not classified as community-based outreach, and studies lacking relevant outcome measures. Additionally, studies with poor methodological quality or inadequate reporting were excluded.

The problem of loss to follow-up in non-communicable diseases

Loss to follow-up cases in hypertension, a non-communicable disease condition characterized by elevated blood pressure, pose a significant impediment to effective management and prevention of associated complications. Hypertension, often referred to as the "silent killer," is a global health concern with a high prevalence and a range of risk factors, including lifestyle choices and genetic predisposition. Understanding and addressing loss to follow-up in hypertensive patients is crucial for preventing severe consequences such as heart disease, stroke, and kidney dysfunction [5].

One notable study that sheds light on the issue of loss to follow-up in hypertension is the "Hypertension Detection and Follow-up Program" conducted in the United States in the 1970s. This landmark study aimed to evaluate the effectiveness of community-based interventions in identifying and managing hypertension. While the program demonstrated that systematic blood pressure screening and follow-up care could lead to a significant reduction in blood pressure levels, it also revealed challenges related to loss to follow-up. Many participants failed to adhere to the prescribed follow-up appointments, hindering the long-term success of the intervention [6].

Several factors contribute to the loss of follow-up cases of hypertension. Lifestyle factors, such as lack of physical activity, poor diet, and tobacco use, can exacerbate hypertension and make individuals less likely to engage in follow-up care. Additionally, socioeconomic factors, including limited access to healthcare, financial constraints, and transportation issues, can impede regular check-ups and medication adherence. A study conducted in low-income urban communities highlighted the impact of socioeconomic disparities on loss of follow-up in hypertensive patients, indicating that individuals facing financial difficulties are more likely to discontinue medical care, leading to uncontrolled blood pressure [7].

Cultural beliefs and health literacy also play a role in hypertension management and follow-up. Misconceptions about the chronic nature of hypertension or skepticism regarding the necessity of long-term medication can contribute to patients neglecting follow-up appointments. A cross-cultural study conducted in diverse populations demonstrated variations in beliefs and practices related to hypertension management, emphasizing the need for culturally sensitive interventions to address loss to follow-up effectively [8].

How loss to follow-up was reduced for communicable diseases?

**Figure 2:**
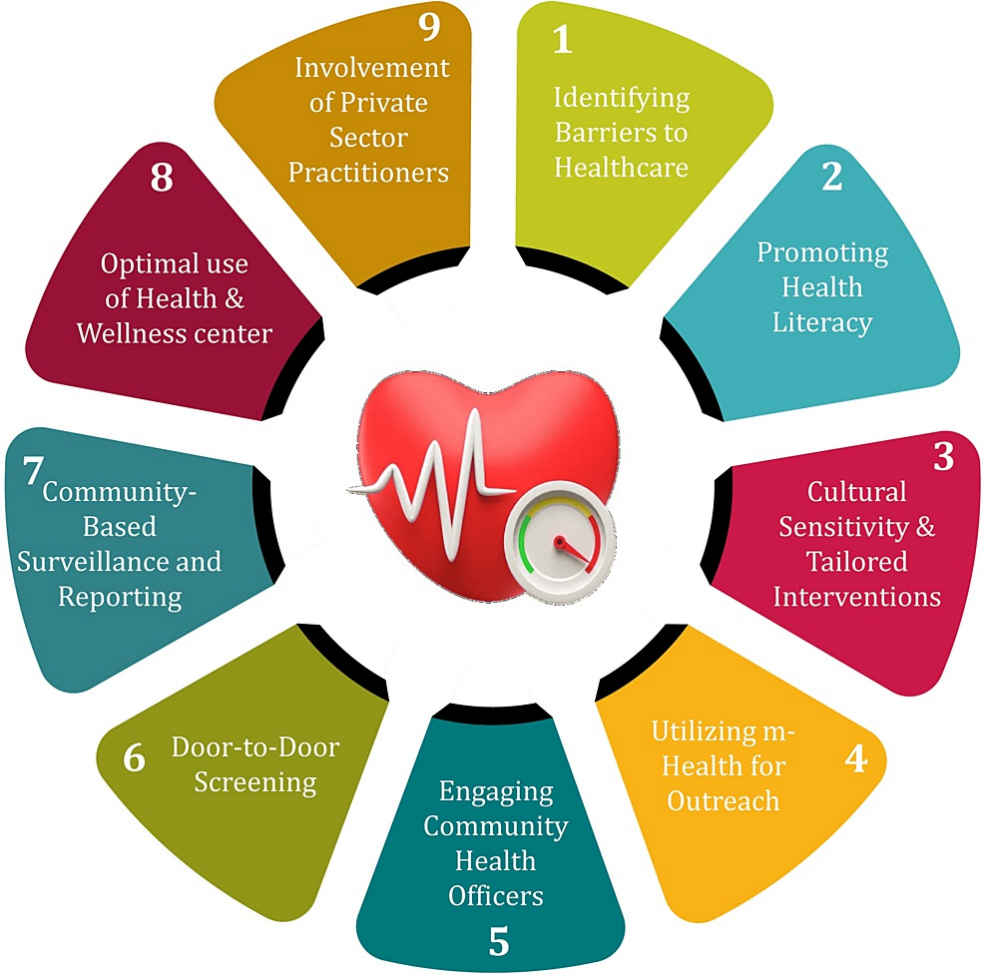
Strategies for controlling hypertension by community-based outreach program Image credits: Sudip Bhattacharya

Addressing loss to follow-up requires a comprehensive and tailored approach. Community outreach programs play a pivotal role in preventing loss to follow-up in communicable diseases by addressing barriers to healthcare access, promoting awareness, and fostering a sense of community engagement. In this comprehensive discussion, we will explore the multifaceted role of community outreach programs in preventing loss of follow-up, drawing examples from various communicable diseases [9, 10]. The themes are summarized in Figure 2.

Identifying Barriers to Healthcare Access

Community outreach programs are instrumental in identifying and addressing barriers that contribute to loss of follow-up in communicable diseases. Barriers such as lack of transportation, financial constraints, and stigma often prevent individuals from accessing healthcare services regularly. An example comes from HIV/AIDS outreach programs in sub-Saharan Africa. In regions where transportation infrastructure is limited, mobile clinics and community health workers play a crucial role in reaching remote areas, conducting screenings, and ensuring that individuals living with HIV/AIDS have access to follow-up care [11].

Promoting Health Literacy and Awareness

Education is a cornerstone of community outreach programs. Increasing health literacy and raising awareness about the importance of follow-up care are vital components in preventing loss of follow-up. For instance, in tuberculosis (TB) control programs, community outreach efforts involve educating the public about the duration of treatment, potential side effects, and the significance of completing the entire course of medication. This awareness helps dispel misconceptions and encourages individuals to stay engaged in their treatment plans [12].

Cultural Sensitivity and Tailored Interventions

Understanding and respecting cultural nuances are critical for the success of community outreach programs. Cultural beliefs and practices can significantly impact healthcare-seeking behavior. An example can be found in initiatives addressing loss to follow-up in hepatitis B management in certain Asian communities. Cultural competence is essential to develop interventions that align with traditional practices and beliefs, reducing the likelihood of discontinuation of care due to cultural misunderstandings [13].

Utilizing Technology for Outreach

In the era of digital advancements, technology plays a crucial role in community outreach. Mobile health (mHealth) interventions, including SMS reminders and telemedicine, contribute to reducing the loss of follow-up. For instance, in the context of antiretroviral therapy (ART) for HIV/AIDS, text message reminders have been shown to enhance medication adherence and decrease loss to follow-up rates. These interventions are particularly effective in reaching populations with limited access to traditional healthcare services [14].

Engaging Community Health Workers

Trained community health workers act as intermediaries between healthcare facilities and the community, building trust and providing personalized support. In the prevention of loss to follow-up in communicable diseases, community health workers can offer counseling, address concerns, and ensure that individuals are connected to the necessary resources. In the context of maternal and child health, community health workers have played a crucial role in promoting antenatal care and ensuring follow-up visits for vaccinations [15].

Door-to-Door Screenings and Outreach Campaigns

Community outreach involves proactive measures such as door-to-door screenings and health campaigns. These initiatives not only identify individuals at risk of communicable diseases but also facilitate early intervention and follow-up care. For example, in the case of vector-borne diseases like malaria or dengue, community outreach teams can distribute bed nets, educate households on preventive measures, and conduct regular screenings to identify and treat cases promptly [16].

Support Groups, Patient Advocacy Groups, and Peer Networks

Establishing support groups and peer networks within communities can be a powerful strategy to prevent loss of follow-up. Individuals facing similar health challenges can provide emotional support and share practical tips for navigating the healthcare system. In mental health outreach programs, peer support has proven effective in reducing loss to follow-up rates, as individuals are more likely to continue treatment when they feel connected to a supportive community [17].

Addressing Stigma and Discrimination

Stigma and discrimination associated with certain communicable diseases contribute significantly to loss of follow-up. Community outreach programs play a crucial role in challenging and dismantling these stigmas. For example, in programs addressing loss to follow-up in HIV/AIDS, community outreach may involve educational campaigns to dispel myths, reduce discrimination, and foster a supportive environment for individuals living with HIV/AIDS to seek and maintain care [18].

Community-Based Surveillance and Reporting

Empowering communities to actively participate in disease surveillance and reporting enhances the early detection of cases and facilitates timely intervention. In the context of emerging infectious diseases, community-based surveillance supported by outreach programs ensures that suspected cases are reported promptly, contributing to the prevention of loss to follow-up during outbreaks [19].

Integration With Existing Healthcare Systems

Effective community outreach programs are integrated into existing healthcare systems. Collaboration between community outreach teams and healthcare facilities ensures a seamless continuum of care. For example, in immunization outreach programs, coordination with local health clinics is essential to track and ensure follow-up vaccinations, reducing the risk of incomplete immunization schedules [20].

The possible roadmap for improving the control rate of hypertension in India

Community outreach programs play a crucial role in preventing loss to follow-up in hypertension, a chronic medical condition characterized by elevated blood pressure. These programs act as a bridge between healthcare systems and communities, addressing barriers to access, promoting awareness, and fostering community engagement. By tailoring interventions to the specific needs of individuals with hypertension, community outreach contributes significantly to the continuity of care, improved medication adherence, and overall management of this prevalent health issue.

One exemplary study that underscores the importance of community outreach in hypertension management is the "Community-Based Hypertension Improvement Project" conducted in urban areas of India. This initiative aimed to enhance awareness, diagnosis, and follow-up care for individuals with hypertension. Community health workers were trained to conduct door-to-door screenings, provide education on lifestyle modifications, and facilitate access to local healthcare facilities for follow-up appointments. The study revealed a substantial reduction in loss to follow-up rates, indicating the effectiveness of community outreach in connecting hypertensive individuals to ongoing care [21].

Identification and addressing of barriers to healthcare access are paramount in community outreach programs for hypertension. A study conducted in low-income urban communities highlighted the impact of socioeconomic disparities on loss of follow-up. Community outreach efforts in these settings involved targeted interventions to alleviate financial constraints, provide information about available resources, and offer support for transportation to healthcare facilities [22]. The result was a notable decrease in loss to follow-up rates, emphasizing the role of community outreach in mitigating socioeconomic barriers that hinder continuous hypertension care.

Promoting health literacy and awareness is a key component of community outreach programs for hypertension. In a cross-cultural study conducted in diverse populations, differences in beliefs and practices related to hypertension management were evident. Community outreach strategies, in this context, included culturally sensitive educational campaigns, involving local leaders and influencers to convey the importance of regular follow-up care. The study demonstrated that tailored interventions significantly improved health literacy, leading to increased awareness and reduced loss to follow-up rates [23].

Technological innovations have also been integrated into community outreach programs for hypertension. Mobile health (mHealth) interventions, such as SMS reminders for medication adherence and follow-up appointments, have shown promise in improving retention in care. For instance, a randomized controlled trial in a rural setting implemented a mobile phone-based intervention to send automated reminders for hypertension management. The results indicated a significant decrease in loss to follow-up, showcasing the potential of technology-enhanced community outreach in supporting individuals with hypertension [24].

The engagement of community health workers is instrumental in the success of community outreach programs for hypertension. Trained community health workers act as advocates and facilitators, providing personalized support and ensuring that individuals are connected to the necessary resources. A study in a large urban healthcare network demonstrated that comprehensive primary care, including routine blood pressure monitoring, personalized counseling, and timely follow-up facilitated by community health workers, resulted in better patient retention and improved blood pressure control [25].

Cultural sensitivity and tailored interventions are essential in community outreach for hypertension, as beliefs and practices regarding health can vary widely. In a study addressing loss to follow-up in hypertension management, cultural competence played a pivotal role in the development of interventions that aligned with traditional practices and beliefs. Community outreach programs in this context involved collaboration with local community leaders, incorporating culturally sensitive messaging, and utilizing traditional communication channels to reach individuals at risk of loss to follow-up [26].

Door-to-door screenings and outreach campaigns are proactive measures within community outreach programs that can significantly impact loss to follow-up in hypertension. In regions with limited healthcare infrastructure, community outreach teams conduct screenings, distribute informational materials, and educate communities about the importance of regular blood pressure monitoring. These initiatives not only identify individuals with hypertension but also facilitate early intervention and follow-up care, preventing loss of follow-up [27].

Support groups and peer networks established within communities contribute to preventing loss to follow-up in hypertension. These networks create a supportive environment where individuals facing similar health challenges can share experiences and provide emotional support. In the management of chronic conditions like hypertension, peer support has proven effective in reducing loss to follow-up rates, as individuals are more likely to adhere to treatment plans when connected to a supportive community [28].

Community-based surveillance and reporting contribute to preventing loss of follow-up by empowering communities to actively participate in disease monitoring. In hypertension management, this involves educating communities about the importance of regular blood pressure checks, encouraging self-monitoring, and reporting abnormal readings to healthcare providers. Community-based surveillance ensures early detection of cases and facilitates timely intervention, reducing the risk of loss to follow-up.

Controlling hypertension necessitates a multifaceted strategy that involves strengthening various components of the healthcare system. First and foremost, a focus on primary healthcare centers is crucial. These centers serve as the front line in healthcare delivery, and by enhancing their capacity to diagnose, manage, and provide continuous care for hypertension, we can ensure early detection and intervention. Additionally, integrating hypertension screening and management into routine primary healthcare services can contribute to better population-wide control [29].

Health and wellness centers play a pivotal role in preventive healthcare. By incorporating hypertension awareness campaigns, regular check-ups, and lifestyle modification programs, these centers can promote healthier living and contribute significantly to hypertension prevention. The emphasis should be on creating an environment that fosters health promotion and disease prevention, making it easier for individuals to adopt and sustain healthy lifestyle choices [30].

Furthermore, the capacity building of Community Health Officers (CHOs) is essential. These frontline health workers can play a key role in hypertension control by conducting community-level screenings, providing education on risk factors, and ensuring adherence to medication regimens. Training CHOs in the latest guidelines, diagnostic techniques, and counseling strategies equips them with the knowledge and skills needed to address hypertension effectively within communities. In a BMJ study, it was concluded that Community Health Worker (CHW)-driven initiatives in the private sector, enhanced by technology, have the potential to systematically identify individuals with non-communicable diseases and successfully provide sustained management for patients with hypertension. Nevertheless, modifications to the existing model, such as integration with the public health system to alleviate financial burdens on individuals, may be necessary to boost its acceptance and consequently enhance its cost-effectiveness [31].

In addition to this, there is a need to involve private sector practitioners in comprehensive hypertension management. An Indian study was conducted with the aim to assess the status of hypertension control and evaluate the quality of care related to hypertension provided to patients seeking healthcare in the private sector. The study revealed that 43.9% (n=265) of patients achieved optimal control of hypertension [32]. Another research project, the Mumbai Hypertension Project, was designed to implement a standardized hypertension management package within private sector clinics located in urban slums. The study documented that despite the implementation of various quality-improvement interventions in private sector clinics in Mumbai, the issue of high loss to follow-up persisted. Blood pressure control rates saw improvement only among patients who adhered to follow-up, without an overall enhancement. The study concluded that to make a substantial contribution to national hypertension control goals, it is essential to introduce new systems that effectively organize and incentivize patient follow-up in the Indian private sector [33].

The role of social media and model digital trends is increasingly significant in elevating awareness about hypertension and mitigating loss to follow-up in healthcare programs. Social media platforms provide a dynamic and accessible avenue for disseminating information, fostering community engagement, and promoting health literacy. By leveraging targeted campaigns and visually engaging content, these platforms can effectively reach diverse demographics, raising awareness about the importance of hypertension management and encouraging regular follow-up. Additionally, digital trends, such as mobile health applications and virtual support communities, contribute to patient empowerment and facilitate continuous monitoring [34, 35].

Acknowledging limitations in data, especially in regions with scarce information on hypertension and loss of follow-up, is crucial. Inadequate data collection, limited healthcare access, and cultural influences may lead to underreporting and bias. Addressing these challenges is essential for a nuanced interpretation. Efforts should concentrate on enhancing data collection, healthcare accessibility, and considering cultural nuances for accurate information on hypertension and follow-up rates in diverse populations and regions [36]. The success of hypertension management programs is intricately intertwined with broader health system factors encompassing policy frameworks, funding mechanisms, and institutional capacities. Policy frameworks play a pivotal role in shaping the direction and priorities of health initiatives. Robust policies that prioritize preventive measures, early detection, and effective management of hypertension contribute to the success of programs [27].

## Conclusions

Community outreach programs may play a vital role in preventing loss to follow-up in hypertension. By addressing barriers to access, promoting awareness, leveraging technology, engaging community health workers, and incorporating cultural sensitivity, these programs contribute significantly to the success of hypertension management initiatives. The examples provided illustrate the diverse strategies employed in different contexts, emphasizing the need for tailored and culturally sensitive approaches to effectively prevent loss to follow-up and improve health outcomes for individuals with hypertension.
